# Degassing during quiescence as a trigger of magma ascent and volcanic eruptions

**DOI:** 10.1038/srep18212

**Published:** 2015-12-15

**Authors:** Társilo Girona, Fidel Costa, Gerald Schubert

**Affiliations:** 1 Earth Observatory of Singapore, Nanyang Technological University, 50 Nanyang Avenue, Singapore 639798; 2 Asian School of the Environment, Nanyang Technological University, 50 Nanyang Avenue, Singapore 639798; 3 Department of Earth, Planetary & Space Sciences, University of California, Los Angeles, CA 90095.

## Abstract

Understanding the mechanisms that control the start-up of volcanic unrest is crucial to improve the forecasting of eruptions at active volcanoes. Among the most active volcanoes in the world are the so-called persistently degassing ones (e.g., Etna, Italy; Merapi, Indonesia), which emit massive amounts of gas during quiescence (several kilotonnes per day) and erupt every few months or years. The hyperactivity of these volcanoes results from frequent pressurizations of the shallow magma plumbing system, which in most cases are thought to occur by the ascent of magma from deep to shallow reservoirs. However, the driving force that causes magma ascent from depth remains unknown. Here we demonstrate that magma ascent can be triggered by the passive release of gas during quiescence, which induces the opening of pathways connecting deep and shallow magma reservoirs. This top-down mechanism for volcanic eruptions contrasts with the more common bottom-up mechanisms in which magma ascent is only driven by processes occurring at depth. A cause-effect relationship between passive degassing and magma ascent can explain the fact that repose times are typically much longer than unrest times preceding eruptions, and may account for the so frequent unrest episodes of persistently degassing volcanoes.

Persistently degassing volcanoes release during quiescence up to several hundred tonnes of SO_2_ per day^1^, whereas total gas emissions (~H_2_O + CO_2_ + SO_2_) are at least one order of magnitude larger and can reach values of up to 10–20 kt/day at Etna^2^, Masaya^3^,^4^, or Satsuma-Iwojima^5^. The loss of such a large mass of gas during quiescence is able to depressurize shallow magma reservoirs by up to several MPa (ref. 6), values which are typically considered to be critical for the evolution of volcanoes. This leads naturally to the question of whether passive degassing affects the magma plumbing system dynamics, that is, whether passive degassing can trigger some of the processes that can lead to eruption.

The most common process that is thought to culminate in eruption at persistently degassing volcanoes is magma replenishment in shallow reservoirs^7^. Magma replenishment has been previously considered to be driven by unspecified processes occurring in deep reservoirs (bottom-up dynamics), and hence it is an input parameter for most eruption models^8^,^9^. In contrast, here we show that there is a possible link between passive degassing and magma ascent, that is, the start-up of magma replenishment and pressurization of shallow magma reservoirs can be indeed triggered by the depressurization induced by degassing during quiescence. This is a top-down mechanism in which eruptions occur by processes occurring at shallow levels, as occurs during unloading by edifice or dome collapse^10^.

## Model

**Figure 1:**
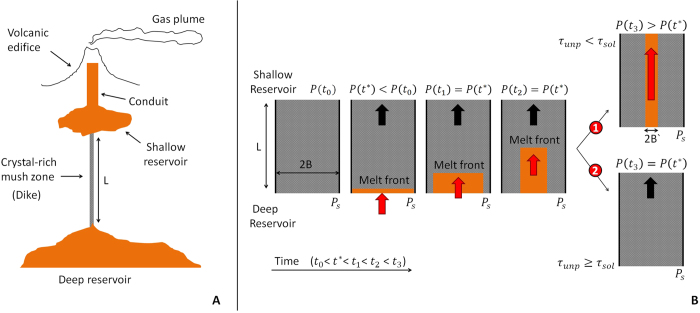
Parts of a persistently degassing volcano and sketch of the magma dynamics in a dike. **(A)** The deep and shallow reservoirs are hydraulically connected through a dike of length *L*. **(B)** Initially (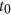) the dike connecting the deep and shallow reservoirs is plugged (grey). The pressure in the shallow reservoir (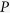) decreases during quiescence, whereas the pressure in the deep reservoir (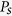) remains constant. After some time (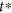), the pressure in the shallow reservoir reaches a critical value (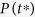), the rigid plug starts to replenish the reservoir, and hotter melt (orange) ascends from the deep reservoir through the dike. During plug removal, the pressure in the reservoir remains constant (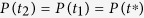) and the melt crystallizes near the dike walls. Two end-member scenarios can take place: (1) the plug is removed before the melt front stiffens, and (2) the melt front stiffens before the plug is removed. When the former happens, pressurization of the shallow reservoir occurs. It is worth noting that a constant value for *Ps* means that the deep reservoir is in equilibrium with its surroundings and thus the pressure *Ps* is always lithostatic. In a more general case, the pressure change in the shallow reservoir during quiescence can be partially transmitted to the deep reservoir and thus *Ps* is not constant either. We also account for this possibility in the text.

To analyse the possible link between passive degassing and replenishment, we take the following into account: (a) persistently degassing volcanoes consist of an open conduit connecting the crater with a shallow reservoir^11^ (~3–10 km depth) (Fig. 1A). Degassing rates are constant on average and the country rock can deform in response to pressure changes^6^. The gas plume is mostly composed of water vapour, and thus passive degassing originates mostly from the shallow reservoir and conduit because H_2_O exsolves at very low pressures. It is worth noting that, even if CO_2_ could come from a deeper reservoir^12^, this would not have an effect on our analysis because of its low proportion in the volcanic plume (see details in ref. 6); (b) the shallow reservoir is hydraulically connected to a deeper reservoir (~10–30 km depth) through a complex interconnected crystal-rich mush zone. This is realistic for frequently erupting volcanoes^13^, and is consistent with the limited seismicity observed during magma ascent and with petrological observations from Merapi^7^, Stromboli^12^, Etna^14^, and Llaima^15^. For simplicity, we model the mush zone as a dike-like structure which is filled with crystal-rich magma during quiescence; (c) the magma filling the dike cools down and crystallizes because of the heat loss to the country rock; (d) the crystal-rich magma is treated as a yield-stress (Bingham) material, such that it behaves as a stiff plug unless the pressure difference between the deep and shallow reservoirs overcomes a certain value^16^,^17^,^18^(Fig. 1B). Pressure changes do not transmit efficiently through yield-stress materials^19^, and thus the pressure variations occurring in the shallow reservoir are not completely transmitted to the deeper one. This creates a pressure difference between both reservoirs.

Based on the aforementioned considerations, the following processes may occur. The pressure in the shallow reservoir decreases during quiescence because of degassing. If the pressure decreases until a critical value, and hence if the pressure difference between the deep and shallow reservoirs increases enough, the stiff plug filling the dike starts to flow upwards (Fig. 1B). The ascent of the plug occurs at constant pressure in the shallow reservoir because depressurization by passive degassing is simultaneously compensated by the pressurization induced by the replenishment of the yield-stress magma (Supplementary Equations S1). Simultaneous with the plug removal, hotter melt with Newtonian rheology flows from the deep reservoir into the dike. The hotter melt cools down and crystallizes near the dike walls while ascending towards the surface, thus decreasing the effective dike thickness^20^ through which the melt can rise. Hence, two scenarios can take place. On the one hand, the crystal-rich plug is removed before the melt front (i.e., uppermost part of Newtonian magma in the dike) stiffens. In such a case, the dike unplugs and the deep and shallow reservoirs are connected through a pathway filled with Newtonian magma. In this scenario, replenishment rate increases and pressurization occurs because the Newtonian melt does not stop flowing when the pressure of the shallow reservoir increases above the critical value. On the other hand, the melt front stiffens before the plug is removed. In such a case, the melt incoming from the deep reservoir becomes a yield-stress material and forms a new plug. In this scenario, only replenishment of crystal-rich magma can occur, and hence passive degassing cannot open connecting pathways to trigger magma replenishment with pressurization. It is worth noting that the condition to open connecting pathways only depends on the thermal evolution of the melt front.

In short, passive degassing can trigger replenishment and pressurization of shallow reservoirs if two conditions are fulfilled. The first condition is that degassing depressurizes the reservoir (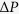) below a critical value (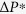), from which the plug starts to flow. This critical value is given by (see details in Supplementary Equations S1):


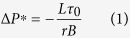


where 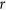 is the ratio of the pressure change in the shallow reservoir that is attenuated by the yield-stress magma filling the dike, and that hence is not transmitted to the deep reservoir, 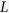 is the length of the dike, 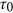 is the yield strength of the crystal-rich magma, and 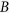 is the half-thickness of the dike. With equation (1) we are obviating the possibility that magma ascent is dominated by uncontrolled overpressures of the deep reservoir, and thus it allows analysing the influence of passive degassing. It is worth noting that the pressure change in the shallow reservoir is completely attenuated if 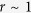. In such a case, the pressure difference between both reservoirs after some time of quiescence is the largest, and is equivalent to considering that the deep magma reservoir is continuously in equilibrium with its surroundings (i.e., the pressure in the deep reservoir is always lithostatic -see Supplementary Equations S1-).

The second condition is that the timescale of dike unplugging (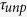) must be smaller than the time needed to stiffen the melt front, that is, the timescale of solidification (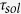). The timescale 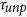 is given by (see demonstration in Supplementary Equations S1):


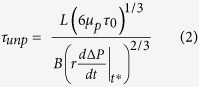


where 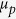 is the so-called plastic viscosity of the yield-stress magma, 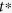 is the time at which the pressure change of the shallow reservoir reaches the value 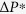, and 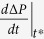 is the depressurization rate^6^ of the shallow reservoir at time 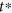. Equation (2) shows that 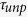 depends on the size of the dike, on the crystal-rich magma rheology, on the efficiency to transmit pressure changes through yield-stress materials, and on the depressurization rate induced by passive degassing. This equation shows that the larger the gas flux and the lower the pressure change transmitted through the Bingham magma, the faster the connecting dike will be unplugged.

On the other hand, the timescale of solidification 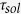 can be estimated by assuming that the cooling of the melt front is dominated by the conductive heat transfer between the melt and the dike walls. In such a case^20^:


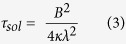


where 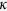 is the thermal diffusivity of the magma and 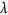 is a numerical parameter that must be determined by solving the transcendental equation 
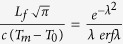
, where 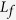 is the latent heat of fusion, 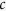 is the specific heat of magma, 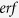 is the error function, 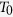 is the initial temperature of the dike walls, assumed to be independent of depth, and 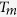 is the initial temperature of the Newtonian magma, assumed to be its melting temperature. In our approach, the melting temperature is defined as the temperature below which the crystal content of the magma is large enough as to cause a transition from Newtonian to Bingham rheology, which can occur for crystal contents as low as 8–20 vol % [ref. 17]. Equation (3) is obtained by solving the so-called Stefan problem, which is justified by the fact that: (a) the front is not affected by the previous passage of melt. Thus, heat advection does not have an influence on the thermal evolution of the front; (b) the temperature that the front ‘feels’ in the walls is always constant (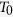) because significant heating does not occur before the passage of the Newtonian melt. The thermal evolution of the entire dike is much more complex because advection and changing temperature of the walls become important^21^ for the magma below the front. However, it does not affect the critical conditions to open connecting pathways and further analysis is beyond the scope of this paper. The details to obtain Equation (3) and the aforementioned transcendental equation for 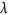 can be found in sections 4.19 and 4.18, respectively, of ref. 20.

## Results

**Figure 2:**
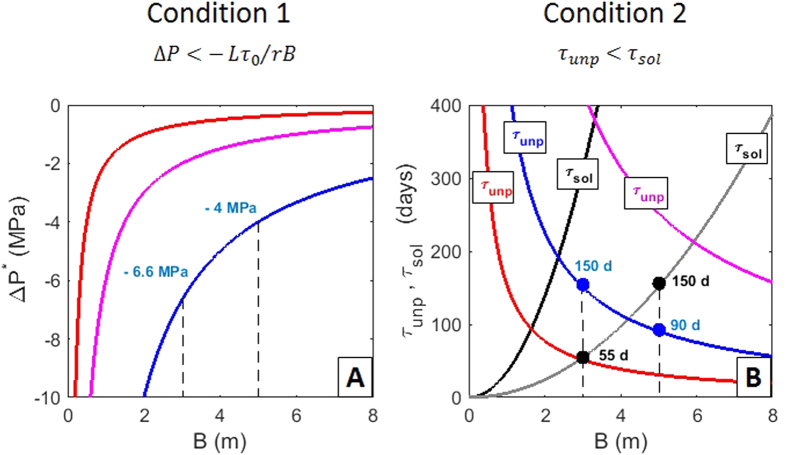
Conditions for magma ascent and pressurization because of passive degassing. **(A)** Critical depressurization 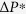 of the shallow reservoir versus the dike half-thickness *B* for three scenarios: 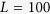 m, 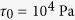, 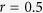 (red); 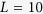 km, 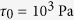, 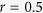 (blue); and 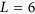 km, 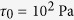, 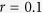 (purple). **(B)** Unplugging (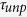) and solidification (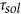) timescales for the same scenarios of panel (**A**). To determine 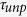, we assume: 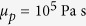, 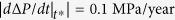 (red); 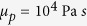, 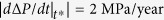 (blue); and 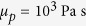, 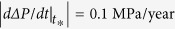 (purple). To determine 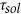, we assume: 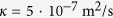, 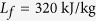, 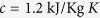., 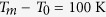 (black), and 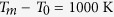 (grey).

We use equations (1)–(3), 12, 20 to analyse whether the conditions 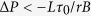 and 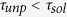 are fulfilled for realistic values of the physical parameters (Fig. 2). We explore different scenarios with 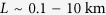 (i.e., the deep reservoir is at most in the upper mantle), 
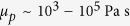
 (i.e., up to one order of magnitude larger than the plastic viscosity measured for basaltic melts^22^,^23^), and 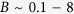 m. This range of values for 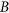 is consistent with the thickness of dikes reported in exhumed plutons^16^, which is used here as a proxy for the thickness of the crystal-rich mush zones connecting the deep and shallow reservoirs. We also consider that 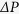 can reach values of up to several MPa during quiescence, with depressurization rates in the range 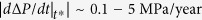 (inferred theoretically for realistic scenarios of persistently degassing volcanoes^6^). The yield strengths of magmas vary over several orders of magnitude. For instance, yield strengths as low as 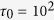 Pa can prevent spreading in the emplacement of laccoliths, whereas values as large as 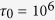 Pa have been proposed for dacite flow^16^,^22^,^23^. For the parameters involved in the heat loss, we consider typical values for the thermal diffusivity (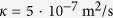), latent heat of fusion (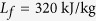), and specific heat of magma (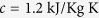). The actual values of 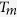 and 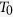 are unknown, thus we use 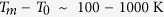 to account for multiple possible scenarios. With low values of 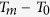, we account for the possibility that the dike walls have been subjected to long-term heating, as could be expected in long-lived volcanic systems. The dependence of 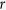 with the properties of the Bingham material and the geometry of the dike is not well constrained, so we analyze different scenarios with 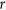 ranging between 0.1 and 1.

For example, for 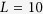 km, 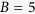 m, 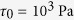,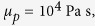 and 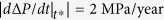, the plug filling the dike starts to flow when 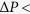 −4 MPa (Fig. 2A), which occurs after 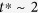 years of passive degassing if we assume that the depressurization rate is constant during quiescence. For this scenario, 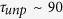 days, whereas 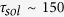 days if 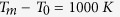
(Fig. 2B). Thus, the plug is removed before the melt rising from the deep reservoir stiffens completely and a connecting pathway can form. The thickness of this pathway can be estimated from^20^ 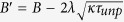, which gives a value of 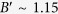 m. For this, we assume as first order of approximation that the thickness of the whole pathway is determined by the final thickness of the melt front. In contrast, a connecting pathway cannot open if 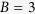 m because 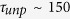 days, whereas 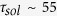 days. We also find that the lower the depressurization rate and ratio of pressure change attenuated, and the larger the plastic viscosity, dike length, and yield strength, the larger the thickness of the dike must be to meet the conditions 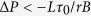 and 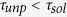. In fact, the conditions are met for a wide range of realistic values of the different parameters if 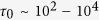 Pa (a range accepted for basaltic-andesitic magmas^16^,^22^,^23^), and even in the case of very low degassing-induced depressurization (~0.1 MPa) (red and purple curves in Fig. 2). Hence, we conclude that passive degassing is able to trigger the opening of pathways to transport hot Newtonian melt to shallower levels, thus increasing the pressure in the shallow reservoir.

The pressure change of the reservoir with time 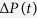 during a cycle of degassing-induced depressurization, dike unplugging, and pressurization can be determined from the set of equations shown in Supplementary Equations S2. These equations correspond to a case in which the reservoir volume is much larger than the conduit volume and passive degassing occurs by means of convection in the conduit^6^. From these equations we can also estimate the volumes of deflation (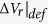) and inflation (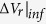) of the reservoir during depressurization and pressurization, respectively. Below, we show a realistic scenario in which the time needed to unplug a dike and pressurize the shallow reservoir is much shorter than the time required to depressurize the reservoir till the critical value 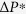. This is consistent with the behaviour of persistently degassing volcanoes since their repose periods are typically much longer (several years) than the unrest periods preceding an eruption (from days to months) (see www.wovodat.org and ref. 24).

**Figure 3:**
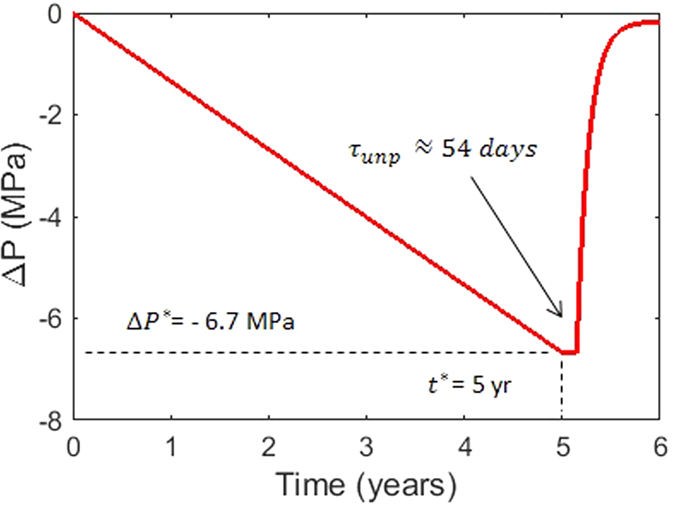
Example of the pressure change with time in the shallow reservoir of a persistently degassing volcano. We use: 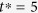 years, 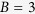 m, 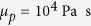, 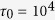 Pa, 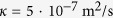, 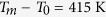, 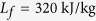, 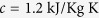, dike width 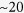 m, gas flux ~ 3 kt/d, conduit radius 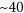 m, reservoir volume 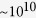 m^3^, bulk modulus of the crust 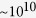 Pa, and effective viscosity of the crust 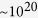 Pa s, mass fraction of volatiles exsolved during degassing 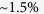, density of degassed magma 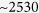 kg/m^3^, density of non-degassed magma 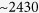 kg/m^3^ (see Supplementary Equations S2 and ref. 6 for details on the calculation of the pressure change with time).

Let us consider that the pressure decreases during quiescence (Fig. 3) until reaching the critical value 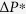 after 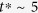 years. By using a realistic total gas flux (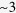 kt/day), conduit radius (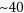 m), and reservoir volume (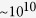 m^3^), we obtain that 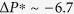 MPa and 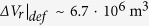. Then, by considering the definition of 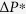 as above, we obtain 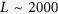 m for 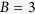 m, 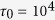 Pa, and 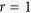 (i.e., the deep reservoir is assumed to be continuously in equilibrium with the hosting rock). Using these parameters, and assuming 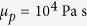, we obtain 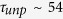 days, whereas 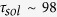 days for 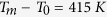. Therefore, a pathway connecting the deep and shallow reservoirs can form with thickness 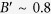 m. After dike unplugging, a very fast magma replenishment and pressurization occur with an initial rate larger than 30 MPa/year, recovering ~95% of the pressure in only half a year. It is worth noting that these rapid replenishments play a key role as instigator of new unrest episodes and volcanic eruptions since the fast ascent of magma can trigger convective overturn in the shallow reservoir^25^ and the sudden expansion of volatiles in the plumbing system^26^. In such cases, the pressurization rate at shallow levels could be even larger than the one we have calculated. If no convective overturn and expansion of volatiles is considered, the reservoir volume would continue to increase at decreasing rates to reach 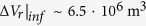 after less than a year.

## Discussion

In this work we have considered pressure changes on the order of up to several MPa, which should induce deformation in volcanic edifices. However, studies based on the analysis of InSAR (interferometric synthetic aperture radar) data at many persistently degassing volcanoes are inconclusive, and no deformation patterns are clearly identified. This could be the result of two factors. First, inflations/deflations of shallow reservoirs of up to ~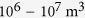 (and thus pressure changes of several MPa according to our model) may not be detected with InSAR data because they are obscured by tropospheric effects. This is the case of, for example, Llaima volcano^27^,^28^. Second, pressure changes during quiescence and prior to eruptions could be indeed very small (<1 MPa), and thus the magnitude and location of the volcanic deformation is difficult to detect. It is worth noting that, even in the case of very low degassing-induced depressurization (~0.1 MPa), it is possible to find a set of realistic parameters that fulfil the conditions to open connecting pathways between a deep and a shallow reservoir.

The pathways that connect the deep and shallow reservoirs are assumed to have a planar geometry (dikes), as constrained from field studies, seismicity, numerical, and analogue experiments^29^. In contrast, the conduit connecting the shallow reservoir with the surface is assumed to be cylindrical, as it stems from direct observations in highly active systems (e.g., Pu’u ‘O’o crater, Kilauea) and from the conical symmetry of many persistently degassing volcanoes (e.g., Mayon, Philippines). The actual geometry of the magma feeding systems is one of the major unknowns in this research field, although planar to cylindrical structures have been also proposed for volcanoes like Soufriere Hills based on the inversion of strain data^30^,^31^. Besides the geometry, other parameters like the yield strength of the Bingham magma or the temperature of the dike walls could also vary with depth. This does not affect the physical mechanism that we propose, and we feel that the unplugging and solidification timescales that we have obtained are feasible because we have covered a wide range of realistic parameters in our analysis.

Another assumption in this study is that cooling of the melt front is dominated by the heat conduction with the dike walls. Heat transfer is also expected to occur between the melt front and the crystal-rich magma right above the front, although it is probably a minor effect because the temperature difference is expected to be much lower than the temperature difference between the melt and the dike walls. On the other hand, degassing-induced depressurization is also expected to change the stress distribution between the deep and shallow storage zones, which could perhaps lead to the opening of new dikes connecting both reservoirs. This would be another mechanism to explain a cause-effect link between passive degassing and the fast ascent of magma towards the surface. However, for very active systems like persistently degassing volcanoes, with yearly to decadal eruptions for hundreds of years, it is likely that the frequent passage of magma between reservoirs has modified the crustal structure. Hence, magma propagation by opening new fractures in the rock seems much less likely than reopening pathways. Finally, it is worth highlighting that the pressure difference between both reservoirs also increases with time at a rate depending on the efficiency at which the pressure changes are transmitted through the yield-stress magma^19^. This efficiency is critical for the pressure balance of magma plumbing systems, and thus exhaustive studies are required in the future to understand how it depends on the geometry, plastic viscosity, and yield strength.

## Conclusions

Our theoretical analysis demonstrates that magma ascent towards the surface and pressurization of shallow magma reservoirs can be triggered by the gas mass lost during quiescence. This is a new top-down mechanism of magma ascent that applies to volcanoes with hydraulically connected plumbing systems, as expected to be the case for frequently erupting volcanoes. We also obtain that dike unplugging and pressurization is much faster than degassing-induced depressurization, which can explain the fact that the time between volcanic eruptions is typically longer than the duration of the unrest episodes. Our results stimulate more in-depth analysis to understand the complex coupling between passive degassing and the dynamics of crystal-rich mush zones.

## Additional Information

**How to cite this article**: Girona, T. *et al*. Degassing during quiescence as a trigger of magma ascent and volcanic eruptions. *Sci. Rep*. **5**, 18212; doi: 10.1038/srep18212 (2015).

## Supplementary Material

Supplementary Information
