# Supplementary material for: Degassing during quiescence as a trigger of magma ascent and volcanic eruptions
Source: Sci Rep. 2015 Dec 15;5:18212. doi: 10.1038/srep18212 (PMC4678897; doi:10.1038/srep18212)
Supplement: Supplementary Information [file srep18212-s1.pdf]

Degassing during quiescence as a trigger of magma ascent and volcanic eruptions

Társilo Girona[1,*], Fidel Costa[1,2], Gerald Schubert[3]

[1]Earth Observatory of Singapore, Nanyang Technological University, 50 Nanyang Avenue, Singapore 639798.

[2]Asian School of the Environment, Nanyang Technological University, 50 Nanyang Avenue, Singapore 639798.

[3] Department of Earth, Planetary & Space Sciences, University of California, Los Angeles, CA 90095.

*Correspondence to: tarsilo.girona@gmail.com. Current affiliation: School of Earth and Atmospheric Sciences, Georgia Institute of Technology, Atlanta, GA 30332.

Section S1:

Description. Supplementary Equations: S1 Timescale of dike unplugging. In this section we develop the complete theoretical demonstration of the time required to unplug a dike filled with crystal-rich stiff magma.

Section S2:

Description. Supplementary Equations: S2 Pressure change in the shallow reservoir with time. In this section we show the equations that we use to determine the pressure and volume change of the shallow reservoir during passive degassing.

S1. Timescale of dike unplugging (τ_{unp})

Let us consider that the pressure in the shallow reservoir decreases with time during passive degassing[6], thus gradually increasing the pressure difference between the deep and shallow reservoirs. This pressure difference tends to displace the magma from the deep into the shallow reservoir through a connecting dike, even though the dike remains blocked due to the yield-stress (Bingham) rheology of the cool and crystal-rich magma. That is, the magma inside the dike forms a stiff plug that can move upwards only if the pressure difference overcomes a certain critical value[16-18]. The critical pressure difference can be determined by taking into account that the stiff plug flows upwards only if $\tau_w(t) > \tau_0$, where $\tau_w(t)$ is the wall shear stress at a given time t and τ_0 is the yield strength. By considering a plane narrow slit, the condition for the magma plug to flow steadily can be written as:

$$\tau_w(t) = \frac{B\,[P_s(t) - P(t) - P_{sr}]}{L} > \tau_0 \quad \rightarrow \quad P_s(t) - P(t) - P_{sr} > \frac{L\tau_0}{B} \qquad (S1-1)$$

where $P_s(t)$ is the pressure in the deep reservoir, $P(t)$ is the pressure in the shallow reservoir, P_{sr} is the hydrostatic pressure exerted on the deep reservoir by the weight of magma inside the dike, and L and B are the length and half-thickness of the dike, respectively. Moreover, $P_s(t)$ and $P(t)$ can be written as $P_s(t) = P_s(t=0) + (1-r)\Delta P(t) + \Delta P'(t)$ and $P(t) = P(t=0) + \Delta P(t)$, where $\Delta P(t)$ is the pressure change (negative) induced by degassing in the shallow reservoir, $\Delta P'(t)$ represents a pressure change in the deep reservoir related to uncontrolled processes, like magma generation at depth, and.r is the ratio of the pressure change that is attenuated by the yield-stress magma filling the dike and that hence is not transmitted into the deep reservoir[19]. If we assume that initially (i.e., after a magmatic eruption) the plumbing system is in equilibrium, it is met that $P_s(t=0) - P(t=0) - P_{sr} = 0$, and hence equation ($S1-1$) can be rewritten as:

$$\tau_w(t) = \frac{B[\Delta P'(t) - r\Delta P\ (t)]}{L} > \tau_0 \quad \rightarrow \quad \Delta P\ (t) < \frac{\Delta P'(t)}{r} - \frac{L\tau_0}{rB} \qquad (S1-2)$$

It is worth noting that, if the deep reservoir remains continuously in equilibrium with the hosting rock (i.e., the pressure in the deep reservoir is lithostatic), it is met that $-(1-r)\Delta P(t) = \Delta P'(t)$, and thus equation $(S1-2)$ becomes $\Delta P\ (t) < -L\tau_0/B$. In such a case, the condition for the magma plug to flow is independent of the efficiency at which pressure can be transmitted through the yield-stress magma. From now on, like we are interested in the feasibility of passive degassing as a trigger of magma ascent, we assume for simplicity that it is always fulfilled that $|\Delta P'(t)| \ll |r\Delta P(t)|$ (and thus $\Delta P'(t) \ll L\tau_0/B$). In such a case:

$$\tau_w(t) \approx -\frac{rB\Delta P\ (t)}{L} > \tau_0 \quad \rightarrow \quad \Delta P\ (t) < -\frac{L\tau_0}{rB} \qquad (S1-3)$$

Equation (S1-3) means that the stiff plug formed inside the dike can only start to move upwards and replenish the shallow reservoir from $t > t^*$, where t^* is the time at which the depressurization induced by degassing in the shallow reservoir is $\Delta P(t^*) \equiv \Delta P^* = -L\tau_0/rB$. The replenishment rate is given by the volume of yield-stress magma that moves into the shallow reservoir per unit time, which can be calculated from the steady state Bingham flow as:

$$\frac{dV_{rep}}{dt} = 0 \qquad if\ \ \tau_w(t) \le \tau_0 \ \ \text{and hence}\ \ \Delta P(t) \ge\ -L\tau_0/rB \qquad (S1-4a)$$

$$\frac{dV_{rep}}{dt} = -\frac{2WB^3r}{3\mu_p L}\Delta P\ (t)\left[1 - \frac{3}{2}\frac{\tau_0}{\tau_w(t)} + \frac{1}{2}\left(\frac{\tau_0}{\tau_w(t)}\right)^3\right] \qquad (S1-4b)$$

$$if\ \ \tau_w(t) > \tau_0 \ \ \text{and hence}\ \ \Delta P(t) < -L\tau_0/rB$$

where μ_p is the plastic viscosity of the yield-stress magma, W is the width of the dike, and it is met that $L \gg W \gg B$. When passive degassing decreases the pressure by more than $\Delta P(t^*)$, the magma plug from inside the dike flows and thus replenishes the shallow reservoir. However, this replenishment tends simultaneously to increase the pressure of the shallow reservoir again and thus tends to preclude the ascent of the plug. Therefore, the depressurization of the shallow reservoir by passive degassing during $t > t^*$ is simultaneously compensated by the pressurization induced by the ascent of the stiff plug, and we can consider that dike unplugging occurs at a constant pressure in the shallow reservoir given by $\Delta P(t) = \Delta P(t^*) + \delta\Delta P$, where $\delta\Delta P \ll \Delta P(t^*)$. This can be also written as:

$$\Delta P\ (t) = \Delta P\ (t^* + \delta t) \approx \Delta P\ (t^*) + \left.\frac{d\Delta P}{dt}\right|_{t^*} \delta t \qquad (S1-5)$$

where $\delta t = t - t^*$ and $\left.\frac{d\Delta P}{dt}\right|_{t^*} \delta t = \delta\Delta P$. By taking into account equation (S1- 5) and considering the expression of $\tau_w(t)$ in terms of the pressure change $\Delta P\ (t)$ [see equation (S1- 3)], we can also write:

$$\tau_w(t) = \tau_w(t^* + \delta t) = -\frac{rB\Delta P\ (t^* + \delta t)}{L} \approx -\frac{rB}{L}\left[\Delta P(t^*) + \left.\frac{d\Delta P}{dt}\right|_{t^*} \delta t\right]$$

$$= \tau_0 - \frac{rB}{L}\left.\frac{d\Delta P}{dt}\right|_{t^*} \delta t \qquad (S1-6)$$

where in the last equality we have taken into account that $-rB\Delta P(t^*)/L = \tau_0$. If we now replace equation (S1-6) in the last expression between brackets of equation (S1-4b), we obtain:

$$\left[1-\frac{3}{2}\frac{\tau_0}{\tau_w(t)}+\frac{1}{2}\left(\frac{\tau_0}{\tau_w(t)}\right)^3\right]\approx 1-\frac{3}{2}\left(1-\frac{rB}{\tau_0 L}\frac{d\Delta P}{dt}\bigg|_{t^*}\delta t\right)^{-1}+\frac{1}{2}\left(1-\frac{rB}{\tau_0 L}\frac{d\Delta P}{dt}\bigg|_{t^*}\delta t\right)^{-3} \qquad (S1-7)$$

For those scenarios in which $\frac{rB}{\tau_0 L}\frac{d\Delta P}{dt}\Big|_{t^*}\delta t \ll 1$ (like the scenarios discussed in Fig. 2b of the main text), the terms between brackets of the right-hand side of the above equation can be expanded up to second order terms by using that $(1-x)^{-n}\approx 1+nx+[n(n+1)/2]x^2$, where $x \ll 1$:

$$\left(1-\frac{rB}{\tau_0 L}\frac{d\Delta P}{dt}\bigg|_{t^*}\delta t\right)^{-1}\approx 1+\frac{rB}{\tau_0 L}\frac{d\Delta P}{dt}\bigg|_{t^*}\delta t+\left[\frac{rB}{\tau_0 L}\frac{d\Delta P}{dt}\bigg|_{t^*}\right]^2\delta t^2 \qquad (S1-8a)$$

$$\left(1-\frac{rB}{\tau_0 L}\frac{d\Delta P}{dt}\bigg|_{t^*}\delta t\right)^{-3}\approx 1+3\frac{rB}{\tau_0 L}\frac{d\Delta P}{dt}\bigg|_{t^*}\delta t+6\left[\frac{rB}{\tau_0 L}\frac{d\Delta P}{dt}\bigg|_{t^*}\right]^2\delta t^2 \qquad (S1-8b)$$

Therefore, equations (S1- 7) can be written as:

$$\left[1-\frac{3}{2}\frac{\tau_0}{\tau_w(t)}+\frac{1}{2}\left(\frac{\tau_0}{\tau_w(t)}\right)^3\right]\approx \frac{3}{2}\left[\frac{rB}{\tau_0 L}\frac{d\Delta P}{dt}\bigg|_{t^*}\right]^2\delta t^2 \qquad (S1-9)$$

If we now replace equations (S1- 5) and (S1- 9) in equation (S1- 4b), we obtain:

$$\frac{dV_{rep}}{dt}\approx\frac{WB^3 r}{\mu_p L}\left[\frac{\tau_0 L}{rB}-\frac{d\Delta P}{dt}\bigg|_{t^*}\delta t\right]\left[\frac{rB}{\tau_0 L}\frac{d\Delta P}{dt}\bigg|_{t^*}\right]^2\delta t^2 \qquad (S1-10)$$

which after neglecting third order terms again becomes:

$$\frac{dV_{rep}}{dt} \approx \frac{WB^4r^2}{\mu_p L^2 \tau_0}\left(\frac{d\Delta P}{dt}\bigg|_{t^*}\right)^2 \delta t^2 \qquad (S1-11)$$

By using that $\delta t = t - t^*$ by definition, we can obtain the replenishment rate of the stiff magma plug as a function of time for our specific problem:

$$\frac{dV_{rep}}{dt} \approx \frac{WB^4r^2}{\mu_p L^2 \tau_0}\left(\frac{d\Delta P}{dt}\bigg|_{t^*}\right)^2 (t-t^*)^2 \qquad (S1-12)$$

By integrating equation (S1-12) we can find a correlation between the total volume of the dike ($2BWL$) and the time needed to extract the magma plug from it (τ_{unp}):

$$\int_0^{2BWL} dV_{rep} \approx \frac{WB^4r^2}{\mu_p L^2 \tau_0}\left(\frac{d\Delta P}{dt}\bigg|_{t^*}\right)^2 \int_{t^*}^{\tau_{unp}+t^*} (t-t^*)^2\, dt \qquad (S1-12)$$

from where we obtain after isolating τ_{unp} that:

$$\tau_{unp} = \frac{L\left(6\mu_p \tau_0\right)^{1/3}}{B\left(r\frac{d\Delta P}{dt}\Big|_{t^*}\right)^{2/3}} \qquad (S1-13)$$

S2. Pressure change in the shallow reservoir with time ($\Delta P(t)$)

The pressure change in the shallow reservoir of a persistently degassing volcano can be determined from the model proposed by Ref. 6, which accounts for the gas fluxes measured with monitoring systems, a cylindrical conduit connecting the upper part of the volcanic edifice with a shallow reservoir, the viscoelastic response of the crust to pressure changes, possible magma density changes in the conduit, the exsolution and expansion of bubbles at depth, and the connectivity between a deep and a shallow reservoir. In such a case, the pressure change $\Delta P(t)$ can be calculated from:

$$\frac{d\Delta P\ (t)}{dt} = \frac{C_1 + C_2 \Delta P\ (t)}{C_3} \qquad (S2-1)$$

where C_1, C_2, and C_3 are three constants. For the simpler scenario in which the shallow reservoir volume is much larger than the conduit volume, there is no significant amount of bubbles in the shallow reservoir, and the melt density in the conduit remains constant (i.e., passive degassing occurs by means of convection in the conduit), C_1, C_2, and C_3 can be calculated from[6]:

$$C_1 = -\frac{\hat{Q} + \lambda_d \Delta P_s \rho_w}{\rho_w} \qquad (S2-2)$$

$$C_2 = -\frac{V_r + \lambda_d \mu_c}{\mu_c} \qquad (S2-3)$$

$$C_3 = \frac{\pi R^2 k + g\ \hat{\rho}_{m,c} V_r}{g\ \hat{\rho}_{m,c} k} \qquad (S2-4)$$

where $\hat{Q}$ is the mean gas flux, λ_d is the hydraulic strength of the dike, ΔP_s is the overpressure of the deep reservoir, ρ_w is the partial density of water dissolved in anhydrous melt, V_r is the volume of the reservoir at time t = 0, μ_c is the effective viscosity of the crust, k is the effective bulk modulus of the crust, R is the radius of the conduit, g is gravity, and $\hat{\rho}_{m,c}$ is the mean density of melt in the conduit. The more general solution of equation (S2 - 1) is:

$$\Delta P\ (t) = -\frac{C_1}{C_2}\left(1 - e^{\frac{C_2}{C_3}(t-t_0)}\right) + \Delta P_0 e^{\frac{C_2}{C_3}(t-t_0)} \qquad (S2-5)$$

For the case of passive degassing during quiescence with a plugged dike, we can consider that $\lambda_d = 0$ and thus $\Delta P_\infty \equiv C_1/C_2 = \hat{Q}\mu_c/\rho_w V_r$, and $\Gamma \equiv -C_2/C_3 = [V_r/\mu_c]\ /[\pi R^2/g\ \hat{\rho}_{m,c} + V_r/k]$. If we use $t_0 = 0$ and $\Delta P_0 = 0$, we obtain:

$$\Delta P(t) = -\Delta P_\infty(1 - e^{-\Gamma\, t}) \qquad for \quad 0 < t < t^* \qquad (S2-6)$$

where t^* is determined from the condition $\Delta P(t^*) = \Delta P^* = -L\tau_0/rB$ (see supplementary material S1). During dike unplugging:

$$\Delta P\ (t) \approx \Delta P^* \qquad for \quad t^* \leq t \leq t^* + \tau_{unp} \qquad (S2-7)$$

And after dike unplugging, $\lambda_d = \lambda'_d$, where λ'_d is the hydraulic strength of the pathway formed after the plug removal. Thus, $\Delta P'_\infty \equiv C_1/C_2 = \left[\hat{Q}\ \mu_c - \Delta P_s \lambda'_d \mu_c \rho_w\right]/[\rho_w V_r + \lambda'_d \mu_c \rho_w]$ and $\Gamma' \equiv$

$-C_2/C_3 = [V_r/\mu_c + \lambda'_d]/[\pi R^2/g\ \hat{\rho}_{m,c} + V_r/k]$. By stating that $t_0 = t^* + \tau_{unp}$ and $\Delta P_0 = \Delta P^*$, we obtain:

$$\Delta P(t) = -\Delta P'_\infty \left(1 - e^{-\Gamma'\,(t-t^*-\tau_{unp})}\right) + \Delta P^* e^{-\Gamma'(t-t^*-\tau_{unp})}$$

$$for \quad t > t^* + \tau_{unp} \qquad (S2-8)$$

It is worth highlighting that equation (S2 - 8) depends on the hydraulic strength of the pathway λ'_d, which in turn depends on τ_{unp} and thus on the depressurization rate during quiescence (see equation (2) of the main text). In other words, equation (S2 - 8) shows the link between passive degassing during quiescence and magma ascent with pressurization of the reservoir. By considering only the elastic deformation as first order of approximation, the deflation $\Delta V_r|_{def}$ and inflation $\Delta V_r|_{inf}$ of the magma reservoir can be estimated from:

$$\Delta V_r|_{def} = \frac{V_r \Delta P^*}{k} \qquad (S2-9)$$

$$\Delta V_r|_{inf} = \frac{V_r\left(\Delta P(\,t_{inf} - t^* - \tau_{unp}) - \Delta P^*\right)}{k} \qquad (S2-10)$$

where t_{def} (= t^*) is the time of quiescence with passive degassing and depressurization, and t_{inf} is the time with inflation and pressurization. The pressure change $\Delta P(\,t_{inf} - t^* - \tau_{unp})$ is calculated with equation (S2 - 8).
